# Hybrid Cellulose–Silica Materials from Renewable Secondary Raw Resources: An Eco‐friendly Method

**DOI:** 10.1002/gch2.201700119

**Published:** 2018-06-19

**Authors:** Leticia Vasquez‐Zacarias, Patricia Ponce‐Peña, Tezozomoc Pérez‐López, Edgar A. Franco‐Urquiza, Guillermo Ramirez‐Galicia, Martha Poisot

**Affiliations:** ^1^ Universidad del Papaloapan Circuito Central 200, Parque Industrial Tuxtepec Oaxaca C.P. 68301 Mexico; ^2^ Universidad Juárez del Estado de Durango Facultad de Ciencias Químicas Av. Veterinaria S/N Circuito Universitario Durango Dgo. C.P. 34120 Mexico; ^3^ Universidad Autónoma de Campeche Centro de Investigación en Corrosión Av. Héroe de Nacozari No. 480, Campus 6 de Investigaciones C. P. 24070 San Francisco de Campeche Campeche Mexico; ^4^ Consejo Nacional de Ciencia y Tecnología CONACyT Centro de Ingeniería y Desarrollo Industrial, Playa Pie de la Cuesta 702 Desarrollo San Pablo Querétaro C.P. 76125 Mexico

**Keywords:** cement‐free building materials, energy efficient materials, hybrid materials, secondary raw resources

## Abstract

Hybrid organic–inorganic materials based on cellulose matrix and silica particles are obtained from wastes of the local paper recycling mill and sugarcane mill as renewable secondary raw materials. The performance comparison of these hybrid materials made from secondary raw materials against the materials made from pure, raw sources is discussed. The Fourier transform infrared spectra show that cellulose features prevail even at 43 wt% silica nanoparticles in the hybrid materials. Such a high content of silica originated from sugarcane bagasse ash and hollow glass microspheres contributes to the high thermal stability of the final composites, as seen by thermogravimetric analysis with very low water absorption. This one‐step approach of biobased hybrid materials represents an excellent way to produce high‐performance materials with high content of inorganic nanoparticles for a wide variety of applications like energy efficient building material completely cement‐free.

## 1 Introduction

The increased preference for “renewable carbon” instead of “fossil carbon” is correlated with our need to reduce nonrenewable resources consumption and greenhouse gas (GHG) emissions.^1^ The Kyoto protocol set up the frame to reach a sustainable future.^2^ After ten years of this protocol ratification in December 2015, the Paris COP21 conference set the aim to keep the world under 1.5 °C temperature rise.^3^ Leadership in Energy and Environmental Design (LEED) certifications, carbon tax and other regulations are driving the demand for increased technical applications of durable biopolymers.

The building industry sector is responsible for high‐energy consumption and resource depletion accelerating the global greenhouse gas emissions delivering environmental damage for today and future.^4^ One of the key elements of sustainable building design is to reduce the consumption of resources by adopting reused and recycled materials in innovative construction materials.^5^

Nowadays, the addition of nanoparticles like nano‐SiO_2_ or nano‐TiO_2_ has been used to create concrete with designed performance.^6^ Nano‐SiO_2_ was found to be more efficient in enhancing strength than silica fume. Nano‐SiO_2_ not only behaved as a filler to improve the microstructure but also as an activator to promote pozzolanic reactions.^7^ Nano‐TiO_2_ containing concrete acts by triggering a photocatalytic degradation of pollutants like NOx, CO, volatile organic compounds (VOCs), chlorophenols, and aldehydes from vehicles and industrial emissions.^8^ One of the main challenges of incorporating nano‐reinforcers like nanotubes or nanofibers into cement‐based materials is the proper dispersion of CNTs/CNFs into the matrix, partly due to its high hydrophobicity and intrinsic strong self‐agglomeration.^9^ Recently, the Presidents of Germanys German Future Prizes 2016 was given to the project C^3^ carbon concrete composite with 140 participating institutes and enterprises led by TU Dresden researchers. Such composites contain high‐performance carbon fiber of Toho‐Tenax Company delivering materials that can save up to 80% of the concrete volume providing slimmer constructions of practically zero maintenance.^10^

The materials based on renewable plant fiber like cellulose–matrix composite show limited interaction between the hydrophilic fibers and matrices of common hydrophobic nature that allows poor interfacial bonding affecting the mechanical performance negatively and reducing moisture resistance that drives long‐term properties.^11^ An improved interfacial bonding can be achieved by several ways like mechanical interlocking, electrostatic bonding, interdiffusion bonding, and chemical bonding.^12^, ^13^, ^14^

An accessible and low‐cost method to obtain hybrid organic–inorganic composite materials follows the percolation approach of the solvent exchange method applied to fibers of cellulose that consist of first forming a 3D template through self‐assembly of the fibers then filling the percolating architecture with a selected polymer. Prior to this method, it was impossible to incorporate cellulose particles into nonpolar polymers without the use of graft‐coupling agents or surfactants.^15^, ^16^

This work has followed the percolating approach for preparing the surface of recycled cellulose fibers donated by the local paper recycling mill applying the solvent‐exchange method for an effective facial interaction with inorganic particles of hydrophobic nature like silicon dioxide or silica contained in sugarcane bagasse ash (SCBA) donated by the local sugarcane mill. The main challenge of this work was to design a new hybrid material of homogenous composition made from nonpure cellulose matrix reinforced with SCBA. The new composites are feasible for use as lightweight building material and the performance comparison with composites made from pure raw materials was registered.

## 2 Results and Discussion

The synthesis and characterization of the composite materials was followed by Fourier transform infrared (FTIR) and X‐ray diffraction (XRD) observing that the secondary raw materials were giving place to the new hybrid material.

### 2.1 Fourier Transform Infrared Spectroscopy

**Figure 1:**
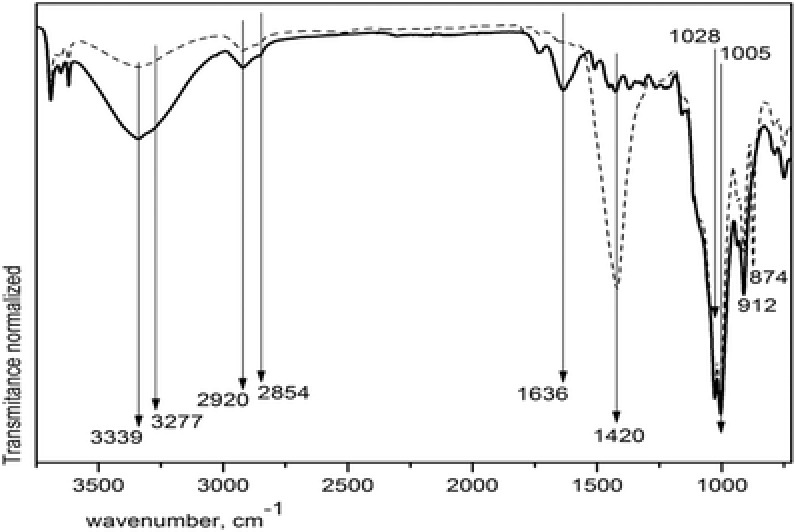
FTIR spectra of paper sludge (dashed line) and after cleaning it (solid line).

In **Figure** **1** it is possible to observe from 3750 to 720 cm^−1^ the most intense absorption peaks of paper sludge and cellulose after paper sludge washing. The band of 3339 cm^−1^ is due to OH frequency region of intramolecular hydrogen bond vibration of secondary alcohol C3—OH…O5 jointly with the 3277 cm^−1^ peak due to the similar vibration of C2 secondary alcohol.^17^ Within the area of 1200–1000 cm^−1^ several absorption peaks are assigned to C—O stretching vibrations from the glucose ring skeletal vibration.^18^

The weak asymmetric vibration of C—O—C is in 1161 cm^−1^ in the glycoside links while the 1106 cm^−1^ vibration is most probably the C—O group, which belongs to the same secondary alcohol as the one at the origin of the OH stretch mode in 3277 cm^−1^. The most intense band in 1028 cm^−1^ is attributed to the C—O group of C6H2—O6H primary alcohols in dominant conformation, while its secondary conformation is observed in 1005 cm^−1^. The next band in 1055 cm^−1^ is also due to C—O group of C3—O3H secondary alcohols.^17^

On the other hand, the strong and wide band of 1420 cm^−1^ is characteristic of asymmetric vibration of CO that is also associated to the strong and wide band of 874 cm^−1^ due to out‐of‐plane angular deformation of group CO_3_ vibration. Both wide bands belong to calcium carbonate in calcite phase, as communicated by several groups that have been studying the calcium carbonate polymorphs.^19^, ^20^ It is evident that the paper sludge as received from the paper mill contains calcium carbonate that is removed with the above‐mentioned reaction with hydrochloric acid solution delivering a clean surface of that short fiber cellulose that is no more useful for making new paper or paper‐derivative products. It is important to say that the CO_2_ evolved from such reaction is trapped in water just to avoid releasing that pollutant to the atmosphere. After cleaning the cellulose pulp the medium band at 912 cm^−1^ is clear, indicating the antisymmetric out‐of‐phase ring stretching of amorphous cellulose.^18^ When this band is broadening it reflects higher frequency of disordered structure resulted by changes in angles around the β‐glycosidic linkages and rearrangements in hydrogen bonds related to deformation modes of COC, CCO, CCH and stretching vibrations in which motion of C5 and C6 atoms is strongly involved. Also, the appearance of a higher frequency shoulder can be related to the presence of two nonequivalent C—O—C bonds.^21^

The vibration bands in the O—H stretching region of cellulose are related to the amount of intra and interchain hydrogen bonds according to several working groups. In Figure 1, the 3604 cm^−1^ band is absent indicating no water absorbed, neither free OH is present since no 3542 cm^−1^ band is observed. A band in 3463 cm^−1^ would correspond to O2—H2…O6 intrachain but it is absent in the same manner that the band of O6—H6…O3′ interchain that would be in 3412 cm^−1^ but the band in 3339 cm^−1^ can be related to O3—H3…O5 intrachain while 3277 cm^−1^ band is related to H bonds in the I β phase of cellulose driving to the conclusion that the clean pulp of secondary raw cellulose can be a mixture of I β and amorphous phases.^22^, ^23^, ^24^

**Figure 2:**
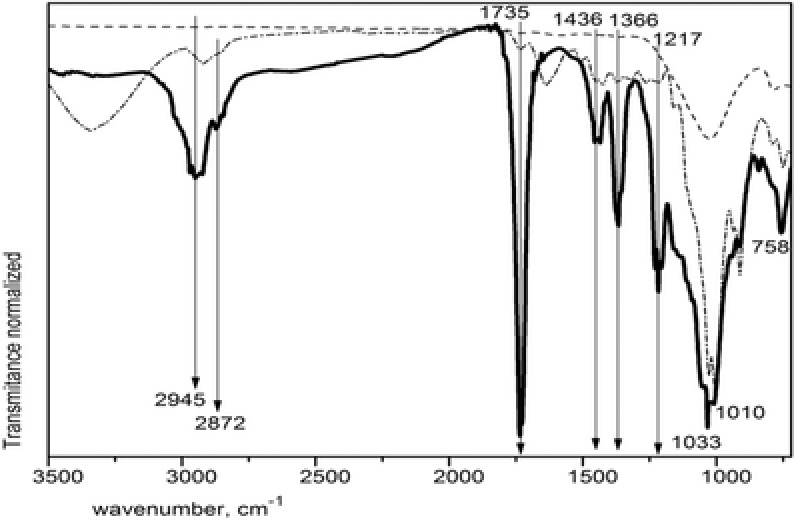
FTIR spectra of hybrid composite agglutinated with RB or CAP (solid line), paper sludge after cleaning (dotted‐dashed line), and dry SCBA (dashed line).

**Figure** **2** shows the spectra from 3500 to 720 cm^−1^ of hybrid composites agglutinated with the commercial acrylic resin against the spectra of after washed paper sludge and dried SCBA that are both the secondary raw materials employed. In the composite spectrum, we can observe four main regions: around 3000, 1735, 1450–1350, and 1250–1000 cm^−1^. In the first region, the bands around 2850 are associated to symmetrical and asymmetrical stretching vibrations of —CH_2_— group of cellulose while the bands around 2900 cm^−1^: 2945 and 2872 are related to vibrations of CH2 and CH3 aliphatic groups of the acrylic resin.^25^ The most intense and fine band in 1735 is assigned to the C=O group vibration from the resin. The weak bands in 1436 and 1366 correlate with the 2900 band due to symmetric vibration. The medium intensity band of 1217 indicated CO group vibration while the wide band of 1092 is related to —O—CH2— axial asymmetric stretching vibration.^26^ Beneath it is the intense band of 1033 with its low shoulder of 1010; such positions are related to C—O group vibrations but also to Si—O—Si group vibrations. The lower intensity bands in 941 and 914 are associated to amorphous cellulose. The absence of 1640 vibration band must be noted since it is related to water adsorption by cellulose.^27^

In summary, we can say that the powders of hybrid composites made from waste cellulose and SCBA agglutinated with acryl‐styrene resin present vibration bands characteristic of cellulose and silica with no content of —OH group vibrations of water, demonstrating a significative apolar interaction between the organic fibers of cellulose and the inorganic particles of silica.

**Figure 3:**
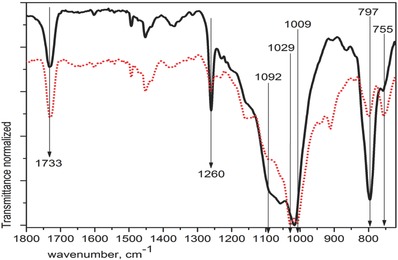
FTIR spectra of black line CP175R and red dotted line CAP1.

**Figure** **3** shows the spectra comparison of CAP1 and CP175R from 1800 to 720 cm^−1^ for clarity. CP175R is the composite made from pure raw materials: C20 and ASi, already agglutinated with acryl‐styrene resin while CAP1 is the composite made from secondary raw resources also containing 1wt% of HGM and agglutinated with the same resin. The spectrum of CP175R shows very sharp vibration bands, just one band in 1017 corresponding to antisymmetric vibration of Si—O—Si in contrast to CAP1 showing two bands: 1009 and 1029. Around 800 cm^−1^, CP175R shows one high intense band in 797 cm^−1^ related to symmetric vibration of Si—O—Si.^28^ Around 755 cm^−1^, the band of CP175R looks very weak while the band of CAP1 shows the same intensity as the band near 800. CAP1 contains more silica than CP175R since HGM is part of the formulation giving place to more than one kind of Si—O—Si vibration modes reflected in two vibration bands close to each other in both regions of 1010 and 800 cm^−1^. On the other hand, CP175R shows in 1092 cm^−1^ a wide band related to Si—O—C vibration because of the high resin content, 60 wt% in contrast to 50 wt% contained in the secondary raw resources composites. Such vibration bands characteristic of Si—O—Si groups were also found by researchers studying hybrid composites of different cellulose matrix: bacterial cellulose^29^, ^30^ or functionalized cellulose acetate^31^ or even lignin matrix.^32^ It is clear from this comparison that CP175R shows the vibration bands of only one kind of silica, pure silica from ASi, in the region of 800 and 1000 while CAP1 shows two bands in both regions indicating that more than one kind of silica is incorporated into the fibers: 300 µm from SCBA and 20 µm from HGM. Such behavior indicates that silica can be incorporated into the cellulose fibers even when the particles are very different in size and the matrix is originated from industrial waste.

Also in CP175R, the medium intense band in 1735 cm^−1^ is assigned to the C=O group vibration from the resin while the fine band in 1260 cm^−1^ is attributed to the attachment of Si—C group vibration onto the silica backbone.^33^ The vibration band extended from 1050 to 1150 can be related to Si—O—C interaction^34^ meaning that the pure raw materials composite, CP175R, and the secondary raw materials composite CAP1 developed good incorporation of the inorganic particles into the organic matrix with higher content of resin in CP175R.

### 2.2 X‐Ray Diffraction

**Figure 4:**
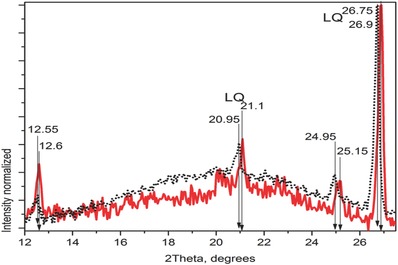
XRD patterns of composites CAP (black dot line) and CAP1 (red line).

The X‐ray diffraction analysis of the cellulose after cleaning the paper sludge shows that calcium carbonate is no more present since the most intense (104) peak is absent in 29.5° indicating that calcite phase, as registered by JCPDS 01‐072‐1650, it has been already washed away with the acid solution reaction.^35^, ^36^ The secondary raw resource washed cellulose looks more like a mixture of two kinds of cellulose: amorphous cellulose with four characteristic features, in 13°, 20°, 26°, and 36°, JCPDS 000601501, and I β phase with maximum in 25° corresponding to the (200) plane, JCPDS 000561718.^37^ The peaks that are found very often in the composites are around 12.5° and 25°. The analysis of SCBA determined the low‐temperature quartz phase (LQ), JCPDS 000050490, the most intense peak is in 26.643° corresponding to the (101) plane while the medium peak in 20.83° belongs to the (100) plane.^38^ These two peaks are identified very often in the hybrid composites XRD analysis. The XRD patterns comparison of CAP and CAP1 within 12°–27.5° can be observed in **Figure** **4**, where the effect of HGM in addition to the composite CAP1 formulation is related to the peaks consistently shifting to higher angle position than in CAP: 12.55° versus 12.60°, 20.95° versus 21.10°, 24.95° versus 25.15°, 26.75° versus 26.9°. Such peaks maximum shifting is presumably corelated to decreasing of lattice plane spacings of the matrix according to the Bragg's law.^39^ In order to clarify that effect a study of X‐ray photoelectron spectroscopy and small‐angle X‐ray scattering is under progress. However, the XRD data of CAP2 and CAP3 show the same trend of higher angle position shifting with the increase of inorganic particles content, remember that CAP1, CAP2, and CAP3 contain HGM in 1, 2, and 3 wt%, respectively.

### 2.3 Thermogravimetric Analysis (TGA)

**Figure 5:**
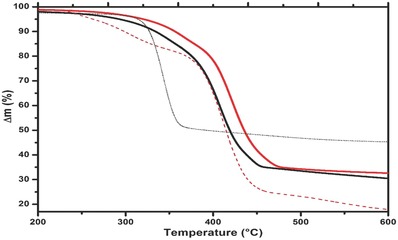
TGA curves of CAP (black line), CAP1 (red line), CP175 (dotted line), and CP175R (dashed line).

In order to determine the silica content in the composites, TGA measurements were carried out in air from 50 to 600 °C. The comparison of thermal degradation of CAP versus CAP1 and CP175 versus CP175R is observed from 200 to 600 °C for clarity in **Figure** **5**. In these composites, the thermal degradation occurs broadly, the shortest one is 250–360 °C in CP175 while the widest one is 220–460 °C in CP175R. CAP shows the interval 250–455 °C while CAP1 shows it from 245 to 475 °C. The composites of lowest resin content absorbed more amount of humidity as registered in CP175 of cero resin content, with 2% humidity released before 200 °C while CAP released 0.85%, CP175R 0.77 °C and CAP1 just 0.47%. A very different behavior is displayed by CP175 in comparison with every other composite since its thermal degradation involves just one step while the other composites including CP175R displayed three events. The highest weight loss takes place in the following order: CP175 in 331 °C, CP175R in 409 °C, CAP in 411 °C, CAP1 in 425 °C, CAP2 in 421 °C, and CAP3 in 423 °C. The corresponding total weight loss (%) for: CP175 is 57, CP175R is 83, CAP is 70, CAP1 is 69, CAP2 is 68, and CAP3 is 66 indicating that CP175 retains the exact amount of silica incorporated in its total formulation, 43%, since no resin is contained. The composites with HGM added have shown higher mass remanent: CAP1 31%, CAP2 32%, and CAP3 34% comparing with, respectively, 21.7, 22, and 22.3% calculated according to the total composite formulation considering resin addition (see **Table** **1**). When we compare such results with CAP that contains no HGM, it is evident that it retained only 8.5 wt% more than the calculated 21.5% but the composites containing SCBA and HGM have shown mass retention of nearly 10 wt% more. Such results indicate that the addition of HGM and SCBA is thermally stabilizing the composites.

**Table 1:** Composites composition of cellulose matrix and inorganic fillers

Constituents wt%	CAS 20 µm	iM16K	RB
CAP	100	0	100
CAP1	99	1	100
CAP2	98	2	100
CAP3	97	3	100
CP175	100	0	0
CP175R	100	0	150

The mass remanent of the pure cellulose composite with no resin added, CP175, is very high, 43%, when compared to pure cellulose decomposition pattern under the same heating rate that has shown the maximum of thermal degradation at 317 °C but the complete thermal event takes from 270 to 340 °C with residual mass of just 4% after 420 °C according to the literature.^40^

The effect of agglutinating resin addition on the pure raw materials composite like CP175R is very significative in comparison with the secondary raw materials composites, the series of CAP. In CP175R, the remanent mass is 17%, just the exact amount of silica contained as calculated, while CAP, CAP1, CAP2, and CAP3 have shown better stabilization of mass against the temperature. It means that the amorphous surface of SCBA is providing an effective interface with the matrix in addition to the thermal shielding effect of HGM. We relate this feature as a consequence of the solvent exchange method applied to the organic matrix. The thermal properties shown by these secondary raw materials composites indicate the feasibility to apply them as thermal insulator of building construction. Recently, in the work of Celebi to prepare composites by melt extrusion method trying to improve the surface interaction between polypropylene (PP) and the same HGM used in our study, the coupling agent of trimethoxysilyl propyl methacrylate (TMSM) was used to modify the surface of up to 20 wt% of HGM added to the final composites. However, no significant interaction was obtained since the thermal conductivity of the composite with 20 wt% of modified HGM increased in 14 wt% compared to neat PP. They explain the poor results obtained due to poor interfacial adhesion between the matrix and the filler.^41^

Another way to obtain pure silica reinforced cellulose composites was performed by the group of Wang delivering a series of composites aerogel with pure cellulose nanofibril aerogel and using tetraethyl orthosilicate (TEOS) as precursor of silica with tailored silica content, 20–70 wt%. Even when the onset temperature of thermal degradation of all composite aerogel was higher than that of pure cellulose aerogel due to the effect of the silica nanoparticles incorporated into the composites aerogel there was no clear correlation between the onset degradation temperature and the increasing silica content. The best thermal stability was observed in just 50 wt% silica content of composite aerogel. They explained that limited nanoscale compatibility between organic–inorganic phases affected negatively the composite aerogels thermal stability.^42^

The data of Lu working group support the synergy effect of the synthetic process on the cellulose–silica composite aerogels obtained by freeze‐drying technology since the silica nanoparticles were chemically bonded on the surface of the cellulose fibers delivering thermal stability, even a reduction of up to 50% of moisture adsorption was observed.^43^

Comparing the results of this work with those obtained in our previous work of hybrid bionanocomposites synthesized by only one step, using only pristine raw materials of cellulose and silica we observed that after 500 °C degradation the composites of solvent exchange treated matrix retained about 12 wt% more than the composite of no solvent exchange treated matrix indicating that the matrix is better stabilized by the reinforcer particles when it is solvent exchange treated delivering higher thermal stabilization to such composites than the composites of just pristine cellulose matrix.^44^ The results of this work indicate that even when the raw materials used are obtained from secondary raw resources the synthesized materials performance is very similar to that of the pristine raw resources composites prepared by a very similar way that is low energy demanding and requires just one‐step synthesis.

### 2.4 Dynamic Mechanical Analysis

Natural fiber (cellulose) reinforced composites have shown good structural damping properties that could be used in the automotive and housing industries.^45^

**Figure 6:**
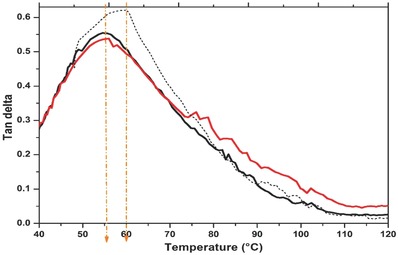
Variation of tan δ of loss factor of CAP (black line), CAP1 (red line), and CP175R (dotted line).

The damping of a composite material may be analyzed by dynamic mechanical analysis (DMA) test. Tanδ is the ratio of the loss moduli to storage moduli or the ratio of energy loss to energy retained during a loading cycle.^46^ Our data registered tanδ from the maximum glass transition temperature (*T*_g_) values that can be seen in **Figure** **6**. The *T*_g_ of CAP and CAP1 is just the same 56 °C, while the loss tangent peak or tanδ differs from each other, 0.56 the first and 0.53 the last one. On the other hand, the composite of pure materials CP175R shows *T*_g_ in 60 °C and tanδ of 0.62. In cases of nanocomposites and filled polymers, increasing the nanoparticle content diminishes the value of tanδ as nanoparticles impose restrictions against molecular motion of polymer chains resulting in more elastic response of the material. Decreasing tanδ means that the material gets more elastic and by applying a load it has more potential to store the load than dissipating it. CAP1 looks like the most elastic of these samples reflecting the effect of incorporation of more than one kind of silica: SCBA and HGM meaning that the matrix and the reinforcers are blending at the microstructure level.^47^ In comparison, CP175R made of pure raw materials shows the highest tanδ indicating poor elastic response even that ASi is contained in this composite.

A very similar behavior was found in the study of composite panels made of natural fiber mats of pure cellulose and recycled paper added with plant‐based oil resin (acrylated epoxidized soybean oil), with 55 wt% of recycled paper the *T*_g_ was in 62 °C while with 20 wt% of pure cellulose *T*_g_ was in 57 °C.^45^

### 2.5 Water Uptake

To test the water uptake of our secondary raw materials composites the series of CAP up to CAP3 were measured demonstrating trend in water absorption by weight percent: 2.8 of CAP, 1.6 of CAP1, 2.1 of CAP2, and 4.3 of CAP3. Such behavior is even lower than natural rubber uptake of 7% after 25 h test^48^ representing clear hydrophobic character of these new hybrid materials. However, the composites made in this study still need further investigation to monitor the degradation resistance over a long period of time (accelerated aging test).

A study of mechanical reinforcement of gelatin hydrogel with nanocellulose as function of percolation concentration considered nanocellulose as reinforcer finding that with 5 g kg^−1^ per 50 g kg^−1^ of matrix the percolation threshold took place.^49^ They explain that the fiber aspect ratio, curliness, and wideness affect the threshold value determination. On the other hand, a recent study to design buoyancy materials with high temperature resistance through a *tert*‐butyl alcohol gel casting process taking borosilicate glass and HGM, the same used by us, but sintering the samples at 650 °C and testing the water absorption they found nearly 40% water absorbed after 10 d due to the open pores observed by scanning electron microscopy (SEM).^50^

The composition of the composites prepared in this study was determined following the percolation approach based on the interconnection in all directions of the phases formed trying to avoid isolated clusters formation.^51^ We can say that the percolation approach threshold of these composites was found by our previous works in 75 wt% of silica based on cellulose matrix when the geometric connection phase occurs as seen by SEM analysis of pure raw materials, while at higher pure silica content the threshold depended on the silica nanoparticle size and shape.^52^, ^53^

## 3 Conclusions

An environmental friendly method was designed after the percolation approach for producing new hybrid materials made from secondary raw materials like renewable short fiber cellulose from the paper recycling mill process and sugarcane bagasse ash from the sugar mill process in comparison to the hybrid materials made from pure raw materials: cellulose and silica nanoparticles. The key step was using the solvent exchange method for no drying the cellulose matrix keeping the voids of biopolymer net to host the inorganic particles. The surface modification of cellulose by this method allowed the observation of its macroscopic hydrophobicity providing compatibility with the inorganic reinforcers. The high amount of silica dispersed into the secondary raw material matrix to deliver the hybrid composites, up to 43 wt% of SCBA, confers characteristics of thermal insulation material to the final composite not only for housing industry application but also for automotive industry purpose since the agglutinating resin used is VOC free.

The best thermal insulator performance was observed in CAP1 due to the effect of not only SCBA but also HGM at the lowest content of 1 wt%.

The method applied in this work is very convenient in the energy and resources aspect as a consequence of using secondary raw materials, avoiding deep cleaning of them and also avoiding chemical modification of the inorganic filler in contrast to several working groups that have tried to disperse the filler into the matrix with no big success applying very energy‐demanding methods. Like a recent report where fumed silica nanofillers, like we used in our present method, were processed with modified poly(lactic acid) observed by transmission electron microscopy (TEM) that particle dispersion into the matrix was not improved.^54^

## 4 Experimental Section

The nanocomposites synthesis take the pure raw materials: silicon dioxide of aerosil 200, 12 nm particle size with 200 m^2^ g^−1^ surface area from EVONIK named ASi, and cellulose fibers of 20 µm provided by Aldrich named C20. The first solvent exchange step was adding water in droplets to the cellulose fibers gently stirring to get a gel during 15 min, then acetone was added in droplets at 1:2 volume ratio according to water volume, stirring was continued for 3 h more. ASi wet with acetone was added in 75 wt% based on cellulose, stirring for 10 min more and 20 min more under ultrasound bath of 40 kHz. Finally, the kind of dough was dried at 60 °C in oven. Such composite was labeled **CP175** and **CP17R** when the powder was agglutinated by addition of acryl‐styren resin Joncryl 1522 from BASF in 60 wt% of the composite total weight resting the mixture for 1 h, then poured into the aluminum cast according to the ASTM C109 standard test method for compressive strength of hydraulic cement mortars,^55^ demolding the piece immediately and drying at 60 °C for 48 h. The four composite samples made from secondary resources took the paper sludge rejected from the paper recycling mill process and the SCBA from the sugar cane mill process. The paper sludge was first washed with mild solution of hydrogen chloride facilitating the following reaction in the cellulose surface

(1) $${\mathrm{CaCO}}_{3}+2\mathrm{HCl}\to {\mathrm{CaCl}}_{2}+{\mathrm{CO}}_{2}↑+H_{2}O$$

The calcium carbonate is remanent of paper additives. The resulting sludge was water washed in order to reach the neutrality of pH, water can be recycled of course and carbon dioxide was just bubbled in water to avoid releasing it to the air. The acetone was added in double volume of the final humidity of the fibers in order to obtain a gel. On the other hand, the SCBA was dried and sieved trough mesh of 300 µm. The powder was wet with acetone for mixing it with the cellulose under mechanical stirring during 3 h and 20 min more under ultrasound bath of 40 kHz, taking care of the temperature to avoid going further than 40 °C. The kind of dough was dried in the oven at 60 °C. The final composition of these composites was kept in 75 wt% of SCBA, based on cellulose. The composites added with fine additive of hollow glass microspheres (HGM) iM16K of 3M, 20 µm particle size, density of 0.49 g cm^−3^ was incorporated in 1, 2, and 3 wt% based on the hybrid composite weight, were labeled CAP1, CAP2, and CAP3 according to the increased content of HGM while the composite with no addition of HGM was labeled just CAP. Every final powder was agglutinated by addition and stirring with acryl‐styrene resin Joncryl 1522 from BASF in no more than 50 wt% of the composite total weight, resting the mixture for 1 h, then poured into the aluminum cast according to the standard ASTM C109, demolding the piece immediately and drying at 60 °C for 48 h.

A total of six hybrid composite samples were labeled as shown in Table 1 and were analyzed by the following techniques: FTIR spectra in attenuated total reflectance mode were recorded by a Perkin Elmer Spectrum 100 with 16 scans per sample. The XRD analysis was measured by a Bruker D8 Advance with Cu anode for 5–50 Theta degree. TGA was performed by a TGA 4000 Perkin Elmer for 5–650 °C at 10 °C min^−1^ under air. Dynamic mechanical analysis was registered in a DMA 8000 Perkin Elmer in a three‐point bending configuration at 1 Hz with heating rate of 2 °C min^−1^ applying a dynamic strain of 0.02%. Specimens were prepared with a nominal length of 50 mm, width of 10 mm, and thickness of 5 mm.

The water adsorption was measured following the ASTM C128 standard test method^56^ when the samples got a constant weight it was considered as the initial weight (*W*_o_). The samples were sunk in water, after 24 h they were dried by paper towels to be weighed again as the final weight (*W*_f_). The water adsorbed in percentage, *W*_a_, was calculated by the following equation

(2) $$\left(\left(W_{f}-W_{o}\right)/W_{o}\right)\times 100=W_{a}$$

## Conflict of Interest

The authors declare no conflict of interest.
